# Supplementary material for: Pool choice in a vertical landscape: Tadpole‐rearing site flexibility in phytotelm‐breeding frogs
Source: Ecol Evol. 2021 Jun 15;11(13):9021–38. doi: 10.1002/ece3.7741 (PMC8258215; doi:10.1002/ece3.7741)
Supplement: Supplementary file 1 — Supplementary Material [file ECE3-11-9021-s001.docx]

**Supplementary Materials**

**Supp. Table 1.** PCA model analysis rank using AIC. All models were coded with a negative binomial family in a GLM framework. Two models fell within 2 AIC of each other; the interaction in the second model was not significant, so we chose the simplest model of the two (Rank 1).


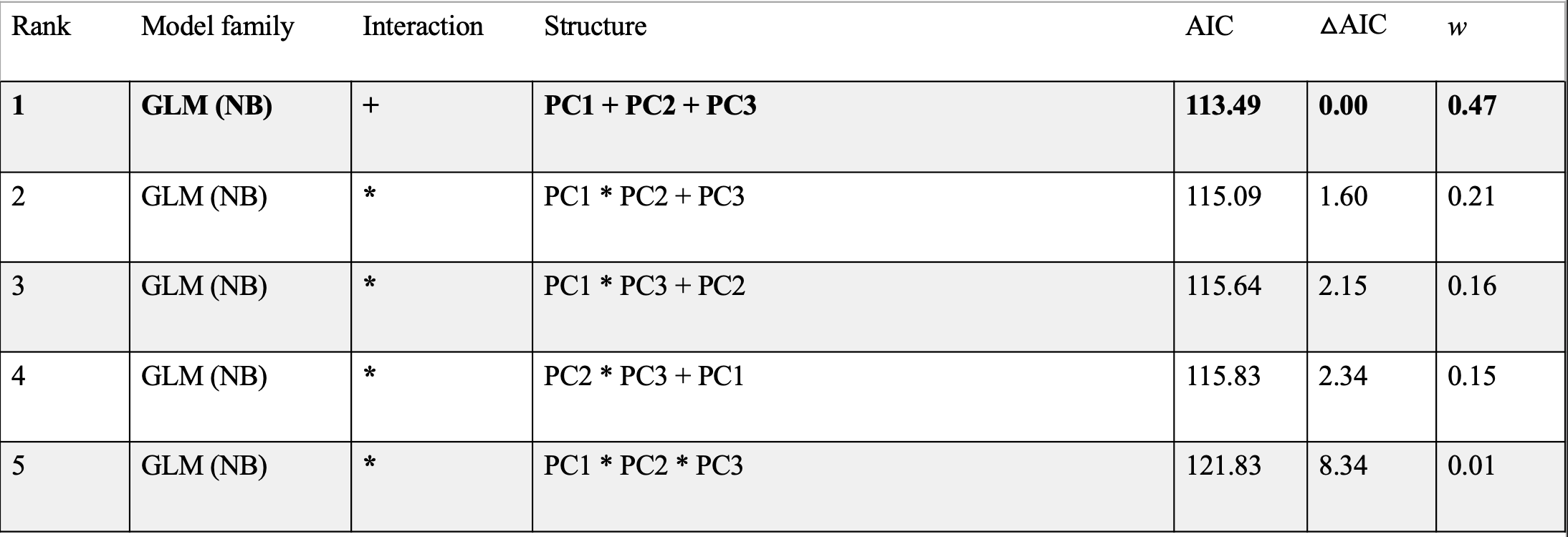


**Supp. Table 2.** **drop1 model selection for the predictors of *pH* measure after repeated pool observations (2020 data)**. Bolded components of each row indicate dropped part of each model iteration. Pool type was a binomial categorical variable (dead/alive), Week indicates week of sampling (numerical variable: 1-4), water capacity was a continuous variables (depth*length*width) of each pool, and Dt_Tadpole_Num was a continuous whole number of *Dendrobates tinctorius* tadpole counts. Interaction term indicated the interaction between covariates. Model family was coded as “Gaussian” for all pH models. Models are ranked in decreasing AIC order. Random effect of pool ID was included in all models.


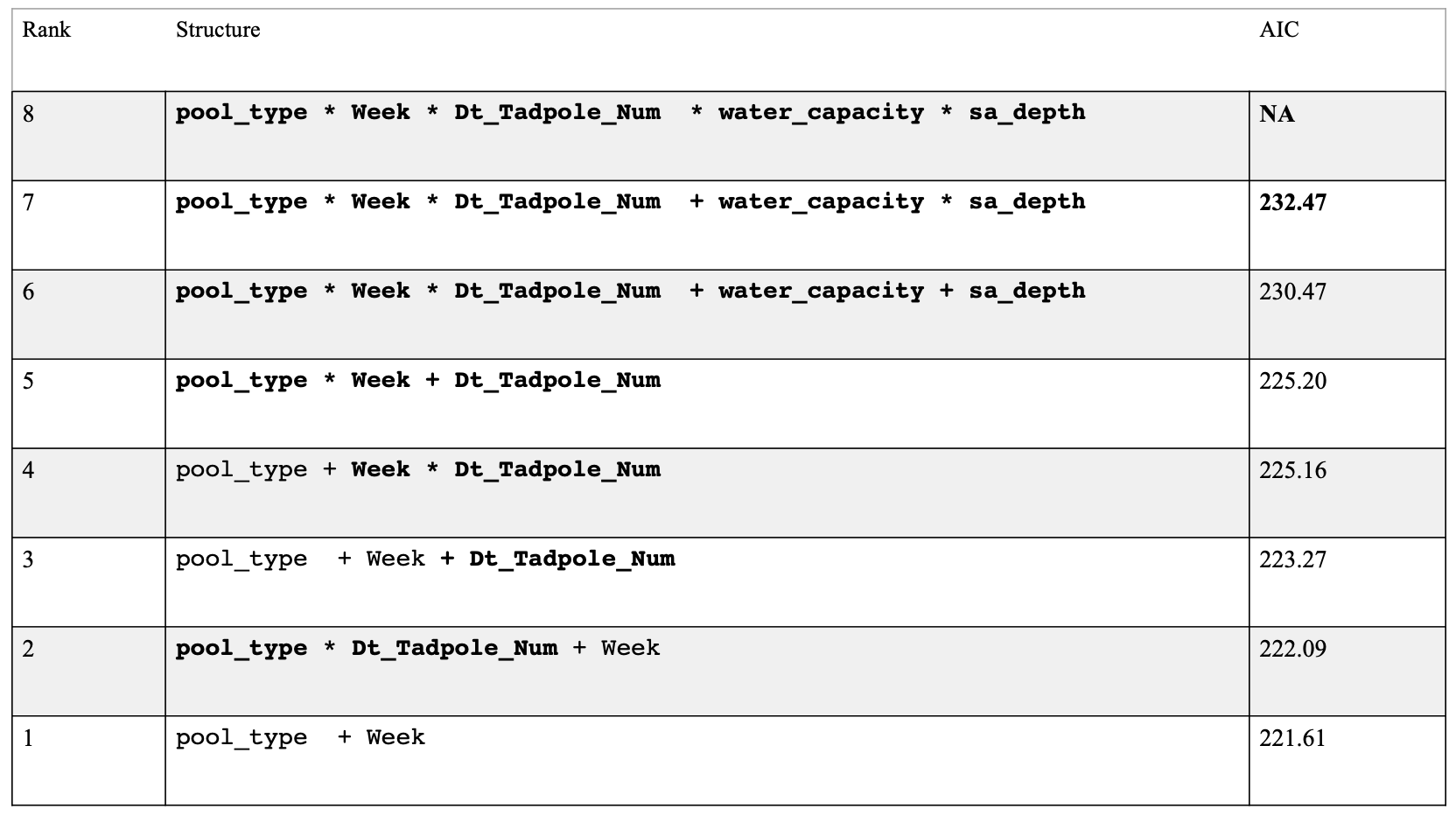


Supp. Table 3. **drop1 model selection for the predictors of *Dendrobates tinctorius* tadpole numbers after repeated pool observations (2020 data)**. Bolded components of each row indicate dropped part of each model iteration. Pool type was a binomial categorical variable (dead/alive), Week indicates week of sampling (numerical variable: 1-4), and pH was a continuous variable, water capacity is pool volume based on semi-ellipsoid equation, and sa_depth is the surface area to depth ratio of each pool. Interaction term indicated the interaction between covariates. Models are ranked in decreasing AIC value. Random effect of pool ID was included in all models. Models were fit with a quadratic (nbinom2) negative binomial family.


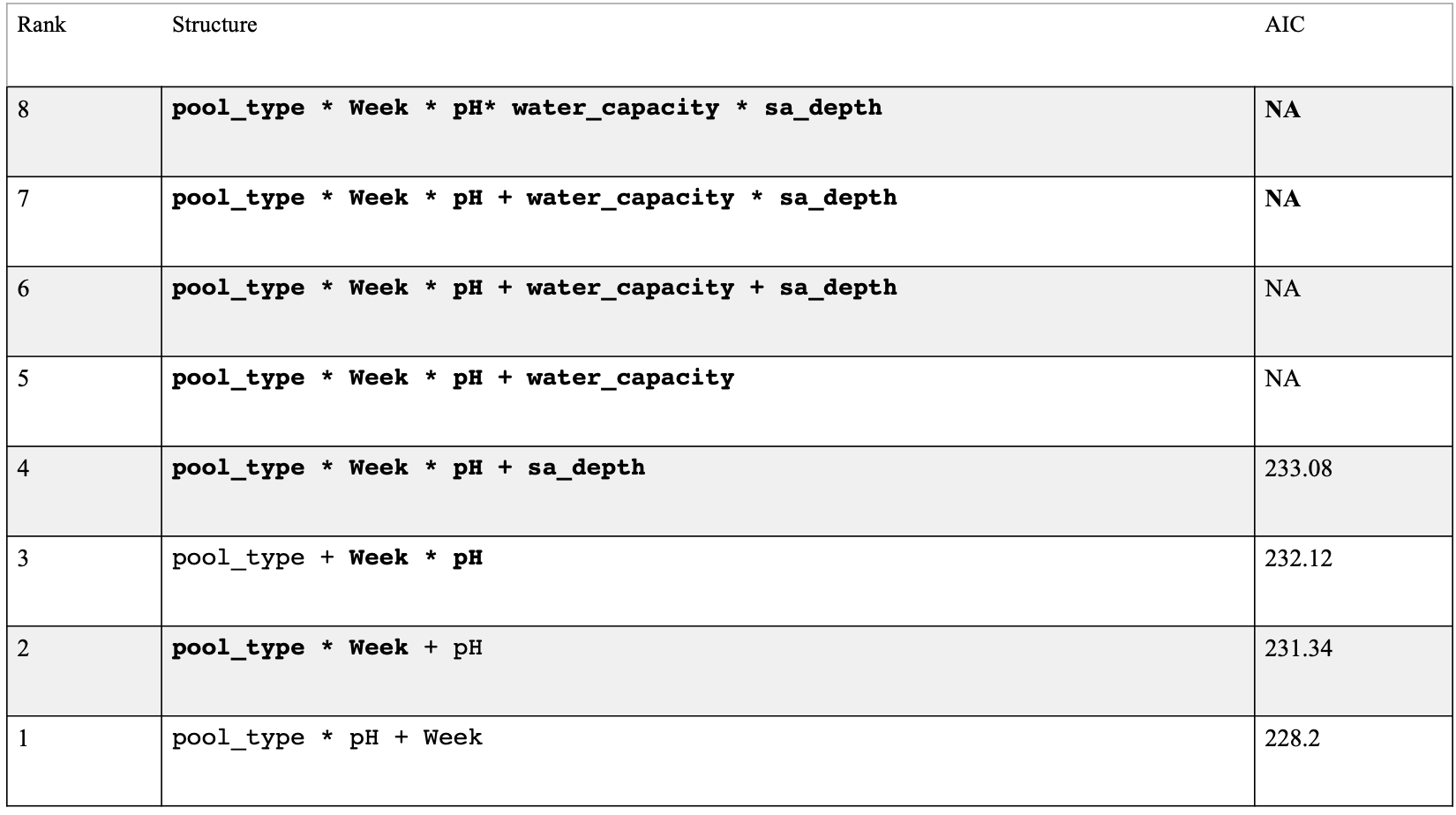


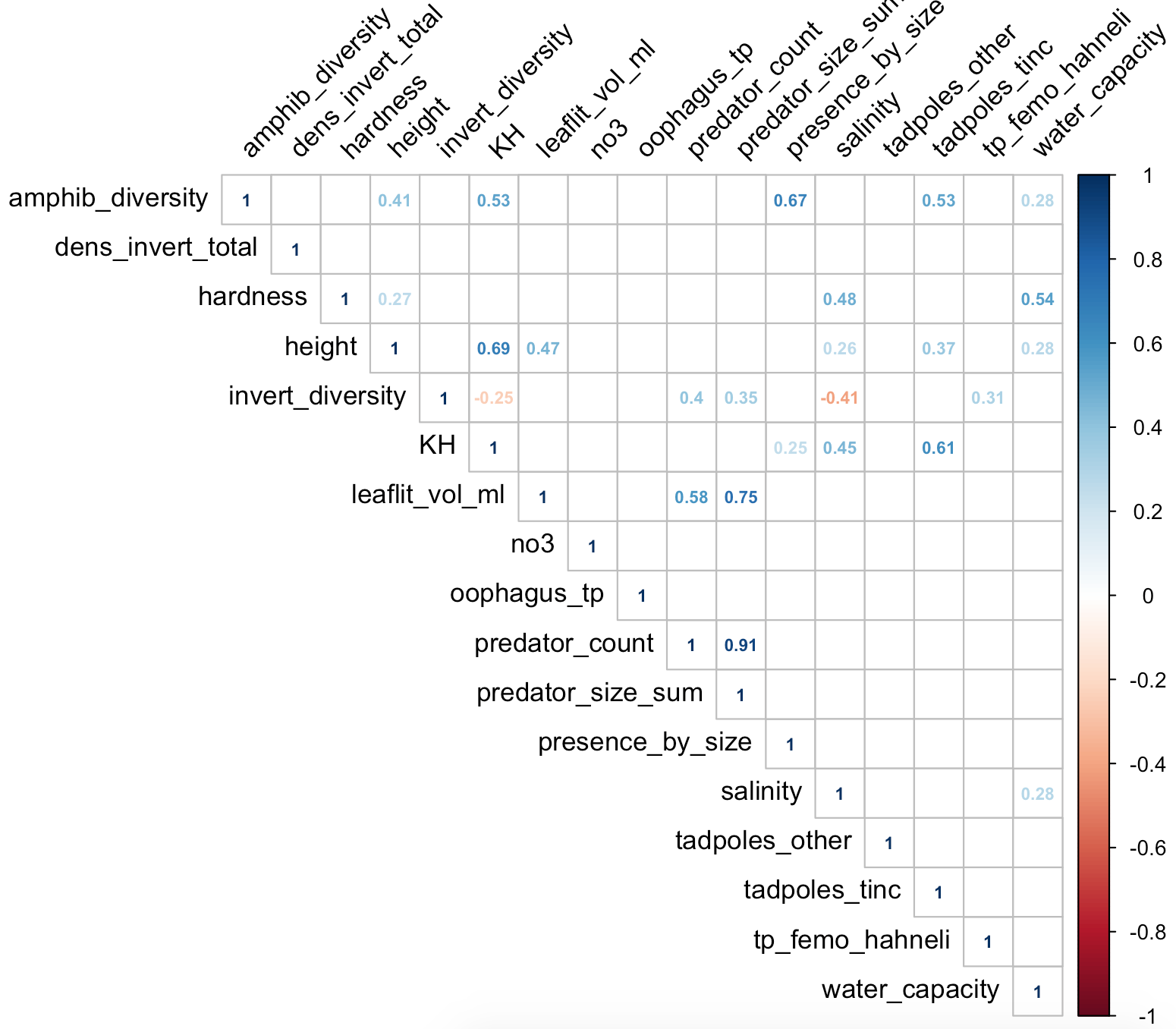


Supplementary Fig 1. High correlation between numeric variables in frogpool data set (2019). Only significant correlations (*p* > 0.05) from a Pearsons’s correlation test are visualized. Cooler colors represent positive correlations and warmer colors represent negative correlations. Variables are ordered alphabetically.


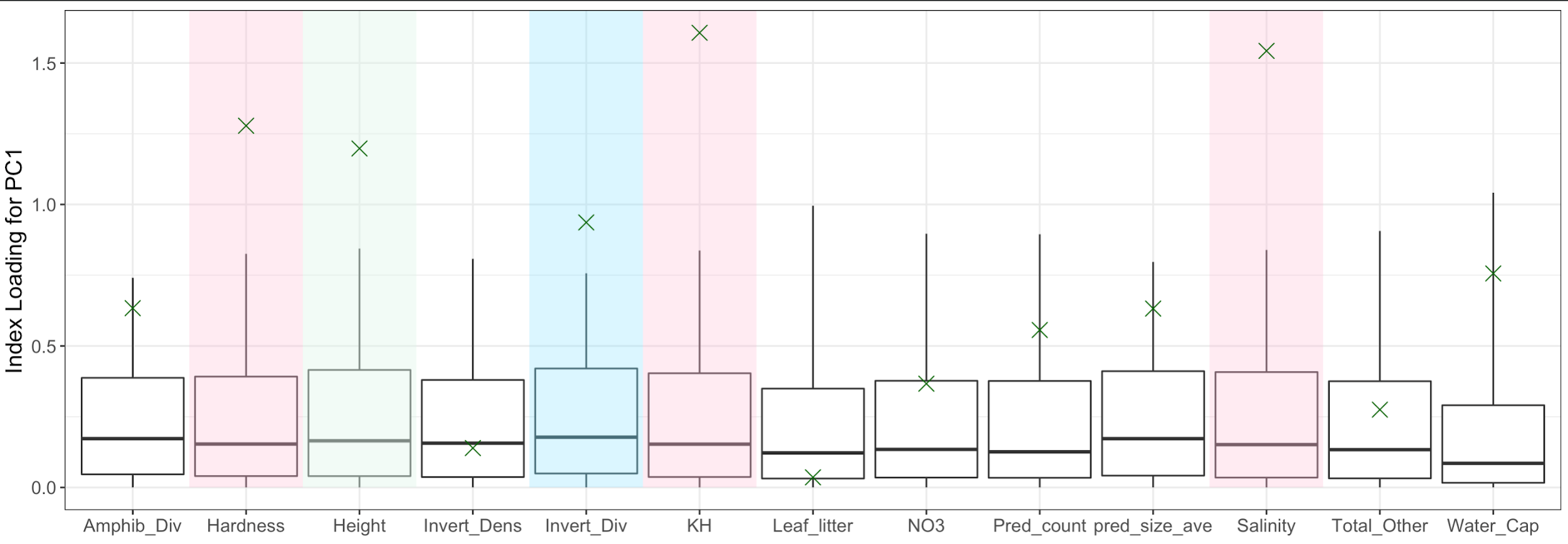


Supp. Figure 2. Results from the PCA correlation procedure. Boxplots are generated from random data, where whiskers range 95% confidence intervals; green “X”s are observed PCA index loading values. Variables where the observed PCA index loading are significantly different from the random confidence interval are highlighted. Pink highlight represents chemical variables, green highlight represents physical variables, and blue highlight represent biological variables.
